# Chronic in utero oxycodone exposure alters placental small EV proteome and fetal cardiomyopathy-linked pathways

**DOI:** 10.20517/evcna.2025.138

**Published:** 2026-02-10

**Authors:** Amin Foroughi-Nezhad, Dalia Moore, Victoria L. Schaal, Tousif Ahmed Hediyal, Elizabeth Stone, Sree Kolli, Pranavi Athota, Omar Shukri, Sowmya V. Yelamanchili, Gurudutt Pendyala

**Affiliations:** ^1^Department of Anesthesiology, College of Medicine, University of Nebraska Medical Center (UNMC), Omaha, NE 68198, USA.; ^2^Department of Genetics, Cell Biology, and Anatomy, College of Medicine, University of Nebraska Medical Center (UNMC), Omaha, NE 68198, USA.; ^3^Child Health Research Institute, University of Nebraska Medical Center (UNMC), Omaha, NE 68198, USA.; ^4^National Strategic Research Institute, University of Nebraska Medical Center (UNMC), Omaha, NE 68198, USA.; ^#^These authors contributed equally to this work.

**Keywords:** Oxycodone, EVs, proteomics, cardiomyopathy, fetal development, EV biomarkers, perinatal exposure, placental signaling

## Abstract

**Aim:** The rising prevalence of opioid use during pregnancy poses serious public health concerns. The placenta is a critical organ during gestation, and opioid exposure can disrupt its function and fetal development. However, the molecular mechanisms through which opioids such as oxycodone affect feto-placental development remain poorly understood. This study aimed to investigate the effects of chronic in-utero oxycodone exposure on the composition and signaling functions of placenta-derived small extracellular vesicles (PSEVs) using a rat model.

**Methods:** Extracellular vesicles (EVs) were isolated from placental tissue and characterized through nanoparticle tracking analysis, transmission electron microscopy, western blotting, and label-free quantitative proteomics. Bioinformatic enrichment analyses were conducted to evaluate changes in EVs biophysical properties and protein cargo.

**Results:** Chronic oxycodone exposure significantly altered PSEV characteristics, including particle size distribution and proteomic composition. Among the 456 identified EV proteins, 107 proteins were significantly dysregulated. We found key downregulatory proteins including Atp2a2, Lmna, Tgfb3, Agt, and Sgce, which are crucial for myocardial calcium cycling, nuclear integrity, extracellular matrix remodeling, and blood pressure regulation. These findings indicate disruptions in fetal cardiac programming, particularly hypertrophic and dilated cardiomyopathy pathways. Additionally, enrichment analyses revealed notable perturbations in metabolic processes (e.g., citrate cycle, fatty acid degradation, N-glycan biosynthesis), along with upregulation of vesicle transport and neurodevelopment-related proteins, indicating broader systemic effects on fetal development. While these proteomic findings are robust, further independent validation (e.g., via targeted assays or Western blotting) will be necessary to confirm individual protein-level changes.

**Conclusion:** These results highlight PSEVs as sensitive molecular indicators linking maternal oxycodone use to disrupted fetal cardiovascular, metabolic, and neurodevelopmental pathways. This study provides a novel systems-level framework for understanding opioid-induced placental signaling alterations and lays the groundwork for developing EV-based diagnostic biomarkers and targeted interventions.

## INTRODUCTION

The global opioid epidemic remains a critical public health crisis, with a troubling rise in opioid use among pregnant women. Among these, oxycodone is frequently prescribed for pain management given its potent analgesic effects^[1]^. However, long-term use of oxycodone can increase the risk of dependency in this vulnerable group^[2]^. Adding complexity, oxycodone readily crosses the placenta, yet its impact on fetal development and placental function remains incompletely understood^[3]^. Increasing evidence links in utero opioid exposure to a wide range of adverse outcomes in offspring - including neurodevelopmental, metabolic, and cardiovascular dysfunction - but the underlying molecular mechanisms remain largely unexplored^[4]^. In particular, how opioids affect the placental microenvironment and its signaling role in fetal organogenesis, especially cardiac development, is a growing area of concern^[5]^.

The placenta is not only a critical interface for nutrient and gas exchange but also acts as an endocrine and paracrine organ, influencing fetal development through extracellular vesicles (EVs). These membrane-bound nanostructures shuttle biologically active molecules, including proteins and RNAs, and serve as molecular messengers between the placenta and maternal or fetal systems^[6]^. Because EVs reflect the physiological or pathological status of their tissue of origin, they are increasingly recognized as potential biomarkers for developmental outcomes in the setting of maternal opioid use^[7]^.

Recent studies have begun to define how placental EVs influence fetal heart development under normal and pathological conditions. For example, Jeyarajah *et al.* (2024) demonstrated in a mouse model that EVs derived from differentiated trophoblast cells are sufficient to promote cardiomyocyte maturation and fetal heart development: fetal hearts exposed to trophoblast-conditioned media exhibited enhanced beat rate, epicardial outgrowth, and increased expression of developmental genes compared to controls. These genes included myosin heavy chain 7 (*Myh7*), natriuretic peptide A (*Nppa*), and NK2 homeobox 5 (*Nkx2-5*) compared to controls^[8]^. These and related works provide strong evidence that EVs are active mediators in the placenta-heart “cross-talk” rather than passive byproducts of placental physiology.

A novel and distinct aspect of our study is the identification of cardiomyopathy-associated proteins within placenta-derived EVs (PEVs) that are altered by maternal oxycodone exposure. Although prior work has shown that EV cargo can mirror placental stress and modulate downstream organ development, no previous studies, to our knowledge, have linked opioid exposure to the coordinated downregulation of a discrete set of cardiomyopathy-related proteins - including Atp2a2, Lmna, Tgfb3, Agt, and Sgce (full names provided in Table 1). These proteins play pivotal roles in cardiac muscle structure, calcium handling, and vascular signaling, and are well known in hypertrophic and dilated cardiomyopathy (DCM) pathophysiology^[9-11]^. By identifying these specific molecular perturbations, our findings offer a mechanistic basis for how maternal opioid exposure may disrupt fetal cardiac programming through EV-mediated signaling.

**Table 1:** Cardiomyopathy-associated genes identified in disease-targeted KEGG pathway enrichment analysis

**Gene symbol**	**Gene name**	**Function summary**
*Atp2a2*	Sarcoplasmic/endoplasmic reticulum Ca^2+^ ATPase 2	Regulates calcium uptake into the sarcoplasmic reticulum, essential for cardiac muscle relaxation
*Lmna*	Lamin A/C	Provides structural support to the nuclear envelope and is linked to cardiomyopathy
*Tgfb3*	Transforming growth factor beta 3	Involved in tissue remodeling, fibrosis, and cardiovascular development
*Sgce*	Sarcoglycan epsilon	Component of the dystrophin–glycoprotein complex; maintains membrane integrity in cardiac muscle

KEGG: Kyoto Encyclopedia of Genes and Genomes.

The placenta and its secreted EVs serve as major conduits for endocrine, immune, and metabolic signals that influence cardiac morphogenesis and vascular development^[8,12]^. Opioids such as oxycodone are known to impair placental vascularization, increase oxidative stress, and dysregulate hormone levels - factors with direct relevance to cardiac developmental trajectories^[9-11]^. In this study, using a well-established preclinical model of maternal oxycodone use^[13]^, we demonstrate for the first time that oxycodone exposure significantly alters EV biophysical properties and protein cargo, with pronounced suppression of cardiomyopathy-associated pathways. The findings of this study highlight the potential of PEVs as molecular indicators of how maternal opioid exposure disrupts cardiovascular and other developmental pathways in the fetus.

## METHODS

### Animals

Male and female Sprague–Dawley rats (8-9 weeks old) were procured from Charles River Laboratories (Wilmington, MA, USA) and housed under standard laboratory conditions with a 12-hour light/dark cycle. Animals had free access to food and water *ad libitum*. All experimental procedures were conducted following the National Institutes of Health Guide for the Care and Use of Laboratory Animals and approved by the Institutional Animal Care and Use Committee (IACUC) of the University of Nebraska Medical Center (Protocol No. 17-080-09-FC).

### Oxycodone treatment

The dosing regimen in this study was derived from previously validated protocols^[14,15]^. Briefly, female rats were divided into 2 groups: Saline and Oxycodone HCL (OXY) treatment (Sigma Aldrich, St. Louis, MO, USA). The female rats were treated with OXY with an ascending dosing procedure, where doses of 10 mg/kg/day of OXY were orally gavage for 5 days, followed by a 0.5 mg/kg/day escalation for 10 days until reaching a final dose of 15 mg/kg/day, after which females were mated with proven male breeders. Similarly, another set of female animals was treated with saline and mated with proven male breeders. At gestational day (GD) 19.5, which is equivalent to the third trimester in humans, animals from both groups were euthanized exactly one hour after the final dose, and placental tissues were isolated. The tissue was then stored at -80 °C for later isolation and analysis of EVs.

To ensure translational relevance, oxycodone dosing was based on validated preclinical models that replicate plasma levels observed in pregnant women undergoing chronic therapeutic or abusive dose exposure^[16]^. The selected dose and frequency achieved steady-state concentrations in rats consistent with clinical pharmacokinetic data. This design emulates maternal exposure scenarios where repeated dosing leads to sustained fetal opioid exposure via placental transfer^[17]^. Studies using similar regimens have shown reproducible behavioral, neurodevelopmental, and physiological outcomes, supporting its validity as a clinically relevant model^[18,19]^. Aligning animal dosing with human data enhances translational interpretation while adhering to ethical limits.

### EV isolation

EVs were isolated from placental tissue as described in prior studies with minor modifications^[20-23]^. Briefly, 450 mg of placental tissue was homogenized and digested in 3.5 mL Hibernate A (HA) containing 20 U/mL papain at 37 °C for 30 min. Enzymatic digestion was halted by adding 6.5 mL of HA supplemented with antipain and a protease inhibitor cocktail. The homogenate was sequentially centrifuged at 300 ×*g* and 2,000 ×*g* for 10 min, followed by 10,000 ×*g* for 30 min at 4 °C to remove residual cells and debris. A 0.22 µm syringe filter was used to filter the supernatant, which was then ultracentrifuged at 100,000 ×*g* for 1 h at 4 °C. The resulting pellet was subjected to density gradient ultracentrifugation using a sucrose step gradient (0.25-2.0 M). EVs were collected from the 0.95 M sucrose layer after 16 h of ultracentrifugation at 200,000 ×*g* and further purified by an additional 1-hour spin at 100,000 ×*g*. Final EV pellets were resuspended in particle-free phosphate-buffered saline (PBS), and protein concentration was quantified using the bicinchoninic acid (BCA) protein assay kit (Thermo Scientific, Waltham, MA, USA) according to the manufacturer’s instructions^[21]^.

To evaluate the quality of the EV preparations and align with Minimal Information for Studies of Extracellular Vesicles 2018 (MISEV2018) guidelines, we included multiple complementary assessments of yield and purity. EV particle concentration was quantified using nanoparticle tracking analysis (NTA; ZetaView, Particle Metrix), and protein content was measured with the BCA assay to calculate the particle-to-protein ratio, a key purity metric recommended by MISEV2018. Preparations consistently demonstrated particle-to-protein ratios exceeding 3 × 10^10^ particles/µg protein, indicative of EV-enriched preparations with a high particle-to-protein ratio, consistent with low soluble protein carryover. Together, these steps provide rigorous validation of EV yield and purity, strengthening confidence in the downstream proteomic and functional analyses.

### Western blot validation of PEVs

To confirm the purity of EVs isolated from placental tissue, western blot analysis was performed using established EV markers and a negative control protein with minor modifications from our previous study^[22]^. EVs protein samples (25-30 µg per lane) were resolved on 4%-12% Bis-Tris gradient gels (Invitrogen, Waltham, MA, USA) under reducing conditions, markers such as Alix and GM130 (cis-Golgi marker) were used, and in a non-reducing condition, the CD81 and CD63 markers were used. Following electrophoresis, proteins were transferred onto nitrocellulose membranes using iBlot2 (Invitrogen) and blocked in 5% non-fat dry milk in TBST. The following primary antibodies were used: Alix (C-11, sc-271975, Santa Cruz Biotechnology, USA), Annexin II (ab214486, Abcam, UK), CD81 (MCA1846, Bio-Rad Laboratories, Italy), and GM130 (610822, BD Biosciences, USA) as a negative control for cellular contamination. Samples were incubated with primary antibodies overnight at 4 °C, followed by horseradish peroxidase (HRP)-conjugated secondary antibodies (anti-mouse, anti-rabbit, or anti-hamster; dilutions ranging from 1:2,500 to 1:5,000) for 2 h at room temperature. Protein detection was carried out using SuperSignal™ West Pico Chemiluminescent Substrate (Thermo Scientific, Waltham, MA, USA), and blots were imaged using the Azure CSeries Imager (Azure Biosystems, Dublin, CA, USA). All procedures followed standardized protocols as described in prior studies and complied with EV characterization guidelines (MISEV2018).

### Transmission electron microscopy acquisition and analysis

Transmission electron microscopy (TEM) was performed to visualize and confirm the morphology of placental EVs, as previously described in studies^[20-23]^. Following isolation, EV pellets were resuspended in 1X particle-free PBS. A 10 µL aliquot of the EV suspension was mixed with 90 µL of TEM fixative solution (2% glutaraldehyde, 2% paraformaldehyde, 0.1 M phosphate buffer). Subsequently, 10 µL of the fixed sample was applied to a copper mesh grid and incubated for 2 min before excess fluid was removed with filter paper. The residual film was allowed to air dry for 2 min. Negative staining was performed using NanoVan, which was similarly wicked off with filter paper and allowed to dry completely. Grids were imaged using a Tecnai G2 Transmission Electron Microscope (FEI, Hillsboro, OR, USA) operating at 80 kV.

### ZetaView analysis

The size distribution and particle concentration of placenta-derived small extracellular vesicles (PSEVs) were assessed using a Zeta View PMX120 nanoparticle tracking analyzer (Particle Metrix, Inning am Ammersee, Germany) with Zeta View software version 8.05.12 SP2, as previously described^[22,24]^. Before analysis, the instrument was calibrated with 100 nm polystyrene nanobead standards. EV samples were diluted 1:10,000 to 1:30,000 in particle-free 1X PBS, and 10 µL of the diluted sample was loaded for measurement. Sample quality and particle drift were evaluated before video acquisition. Videos were recorded at a sensitivity of 65, a shuttle speed of 100, and a frame rate of 30 frames per second. Measurements were taken at 11 positions across three cycles, and final outputs included particle size (nm) and concentration (particles/mL).

### Proteomics analysis

The Pierce BCA Protein Assay Kit (Thermo Scientific, Rockford, IL, USA) was used to determine the protein concentration in the samples as described in previous studies^[15,22,25]^. For mass spectrometry (MS) analysis, 50 µg of protein from control and oxycodone exposed PSEVs were subjected to label-free quantitative proteomics, as detailed in our previously published work^[15,22,25]^ at the Mass Spectrometry Core Facility on campus. Disulfide bonds were reduced by adding 10 mM tris(2-carboxyethyl)phosphine (TCEP) and incubating at 37 °C for 30 min, followed by alkylation of free cysteines using 40 mM acrylamide for another 30 min at the same temperature. Protein precipitation and cleanup were achieved using S-Trap™ microcolumns, which included acidification with 27.5% phosphoric acid and binding in a methanol/triethylammonium bicarbonate (TEAB)-based buffer. To ensure the removal of lipids and other contaminants, each column was washed three times with both 2:1 chloroform:methanol and S-Trap wash buffer.

After trapping and washing, on-column digestion was performed using Trypsin/lysine-C (Lys-C) at an enzyme-to-substrate ratio of approximately 1:12. Digestion proceeded at 37 °C for 2 h, after which peptides were sequentially eluted with 50 mM TEAB, 0.2% formic acid in water, and 0.2% formic acid in 50% acetonitrile. The eluted peptides were pooled, vacuum-dried at room temperature, and reconstituted in 2% acetonitrile with 0.1% formic acid. Peptide concentrations were determined using the Pierce Fluorometric Peptide Assay, following a standard curve generated from a serial dilution of a 1 mg/mL peptide digest standard. For liquid chromatography tandem mass spectrometry (LC-MS/MS), peptides were diluted to a final concentration of 0.2 µg/µL and spiked with indexed retention time (iRT) standards for retention time calibration. Samples (5 µL) were injected onto a µPAC™ (micro-pillar array column) NEO 50 cm nanoflow liquid chromatography (nanoLC) column and separated over a 90-minute gradient using a Thermo Exploris 480 mass spectrometer. The instrument was operated in data-dependent acquisition mode with a spray voltage of 2 kV, precursor ion scan (MS1) resolution of 60,000, fragment ion scan (MS2) resolution of 15,000, and higher energy collisional dissociation (HCD) activation at 30%. Raw files were processed using Proteome Discoverer 2.4 and Spectronaut, with searches conducted against the rat SwissProt and isoform databases. Static modifications included carbamidomethylation of cysteine, and dynamic modifications included oxidation of methionine and N-terminal acetylation. A target false discovery rate (FDR) of 1% was applied at both peptide and protein levels to ensure high-confidence identifications.

### Data analysis

Quantitative proteomic profiles were obtained and analyzed to identify differentially expressed proteins between experimental and control groups. Proteins were filtered based on their log_2_ fold change (log2FC) and statistical significance. Specifically, proteins with log2FC values between +0.58 and +1.00 and *P*-values < 0.05 were classified as moderately upregulated, while those with log2FC values between -1.00 and -0.58 and *P*-values < 0.05 were considered moderately downregulated. These thresholds correspond to a fold change range of 1.5-2 (upregulated) and 1-1.5 (downregulated), commonly used in proteomic studies to detect meaningful biological variation while minimizing false positives.

### Bioinformatics

#### Functional enrichment analysis

Functional characterization of the differentially expressed proteins was performed through Gene Ontology (GO) and Kyoto Encyclopedia of Genes and Genomes (KEGG) enrichment analysis. GO analysis was conducted for biological process. KEGG pathway enrichment was used to identify overrepresented metabolic and signaling pathways. All enrichment analyses were performed using a hypergeometric test framework with multiple hypothesis testing correction via the Benjamini-Hochberg method. Terms with adjusted *P*-values (FDR) < 0.05 were considered statistically significant.

#### Data visualization

For each analysis, the top 20 enriched GO terms and KEGG pathways were selected based on adjusted *P*-value ranking. These were visualized using dot plots and bar plots. In the dot plots, the x-axis represented the negative log10 of the adjusted *P*-value, the y-axis listed the enriched terms, the color gradient reflected significance, and the point size indicated gene count. In the bar plots, bar lengths represented enrichment significance.

#### Biological contextualization

To explore relevance to disease phenotypes, enriched GO and KEGG terms were queried for associations with key cardiovascular and pregnancy-related conditions, including preeclampsia, cardiomyopathy, congenital heart disease (CHD), hypertension, and broader cardiovascular disease. Keyword-based filtering of term descriptions enabled identification of condition-specific enrichment signatures.

#### Gene network analysis

We performed a network-based analysis using GeneMANIA (v3.6.0) with *Rattus norvegicus* as the reference organism to explore functional associations among input genes. GeneMANIA automatically selected the optimal weighting method and integrated data from multiple sources, including co-expression, physical interactions, predicted interactions, pathway associations, co-localization, and shared protein domains. The network was generated by combining the query genes with related genes to improve connectivity, and the resulting interactions were visualized with edges classified by interaction type. Functional enrichment was performed based on GO biological processes, with significance assessed using FDR correction.

#### Statistical analysis

An unpaired *t*-test followed by Welch’s test correction with *P* < 0.05 was performed to identify significant differences between groups (saline *vs.* Oxycodone). All statistical tests related to Zetaview were performed with GraphPad Prism (La Jolla, CA, USA); data are represented as the mean ± standard error of the mean (SEM) on the graphs.

## RESULTS

### Biochemical characterization of PSEVs

**Figure 1:**
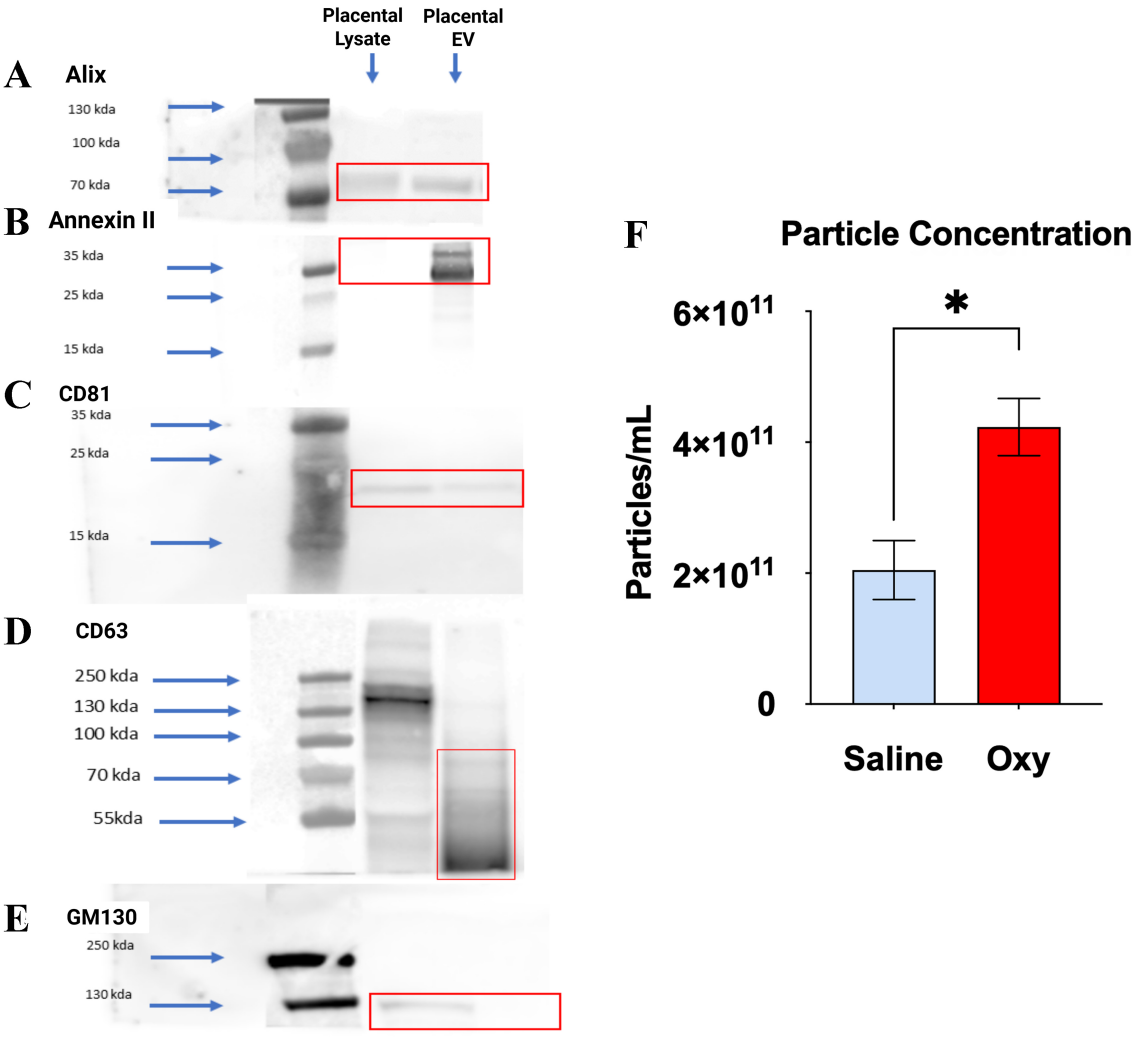
Characterization of placental sEVs via Western blot and particle concentration analysis. (A-D) Western blot analysis demonstrates the enrichment of canonical EV markers in placental EV fractions compared to placental lysate, confirming successful EV isolation. EV samples from both saline- and oxycodone-treated groups were probed for (A) Alix (~90-100 kDa), (B) Annexin II (~36 kDa), (C) CD81 (~25 kDa), and (D) CD63 (~30-60 kDa), all of which showed prominent bands in the EV lanes (highlighted in red boxes); (E) GM130 (~130-250 kDa), a Golgi matrix protein used as a negative control, was detected only in placental lysates and not in EV fractions, indicating minimal cellular contamination. Blue arrows mark molecular weight standards; (F) NTA shows a statistically significant increase in particle concentration in EVs from the oxycodone group compared to saline controls, indicating enhanced EV production. Values are expressed as particles/mL ± SEM. ^*^P < 0.05. Oxycodone exposure alters the morphology and size distribution of PSEVs. An unpaired *t*-test followed by Welch’s test correction with *P* < 0.05 was performed to identify significant differences between groups (saline *vs.* Oxycodone). *n* = 3 biological replicates per group: Saline: *n* = 3, OXY: *n* = 3, [Supplementary Table 1]. sEVs: Small extracellular vesicles; EV: extracellular vesicle; NTA: nanoparticle tracking analysis; SEM: standard error of the mean; PSEVs: placenta-derived small extracellular vesicles; OXY: Oxycodone.

To validate purity of placental small extracellular vesicles (sEVs), we performed Western blot analysis for canonical EV markers and a negative cellular marker. The EV fractions from both saline- and oxycodone-exposed placentas showed strong immunoreactivity for Alix (~90-100 kDa), Annexin II (~36 kDa), CD63 (~30-60 kDa), and CD81 (~25 kDa) [Figure 1A-E], all of which are well-established markers of EVs, confirming successful isolation of vesicle populations. In contrast, the cis-Golgi marker GM130 (~130-250 kDa) - which serves as a negative control for cellular contamination was detected in placental lysates but absent in the EV fractions [Figure 1E], further confirming the vesicular purity of the isolates.

### Oxycodone exposure reduces EV size and alters morphology and concentration profiles

**Figure 2:**
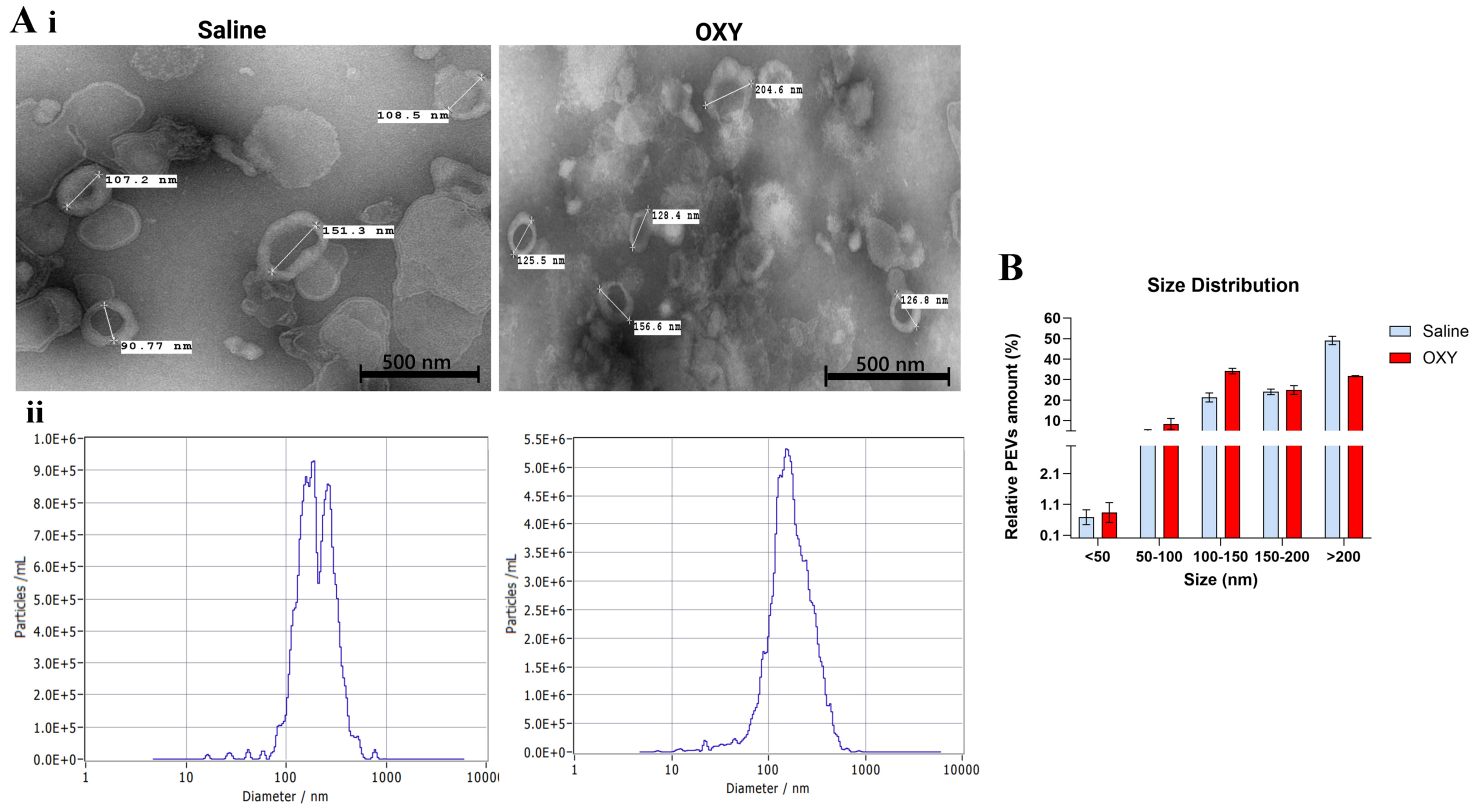
Oxycodone exposure alters the morphology and size distribution of PSEVs. [A(i)] TEM images show representative EVs isolated from placental tissue of saline-treated (left) and oxycodone-treated (right) groups. TEM provides qualitative visualization of vesicle morphology/size. [A(ii)] NTA (ZetaView) profiles show particle size distributions for each group, demonstrating a leftward shift in peak diameter in the oxycodone group, consistent with a higher proportion of smaller EVs; (B) Quantification of EV size distribution across binned size ranges shows increased relative abundance of 100-150 nm EVs and reduced abundance of > 200 nm vesicles in the oxycodone group compared to saline controls. Data are shown as mean ± SEM. Statistical comparisons were performed using an unpaired *t*-test with Welch’s correction. Oxycodone exposure induces differential protein expression in placental EVs. PSEVs: Placenta-derived small extracellular vesicles; TEM: transmission electron microscopy; EVs: extracellular vesicles; NTA: nanoparticle tracking analysis; SEM: standard error of the mean; OXY: Oxycodone.

TEM revealed distinct morphological differences in PSEVs between saline- and oxycodone-exposed groups [Figure 2A(i)]. EVs from control placentas exhibited a typical round morphology with diameters ranging from approximately 170 to 214 nm. In contrast, EVs from oxycodone-exposed placentas were notably smaller, with average diameters around 128-129 nm. The reduced size and altered vesicular structure in the oxycodone group may reflect changes in EV biogenesis, membrane dynamics, or stress-induced vesiculation.

NTA further supported these morphological differences by quantifying the size distribution and concentration of EVs [Figure 2A(ii)]. Oxycodone exposure resulted in a leftward shift in the particle diameter peak, indicating a higher proportion of smaller EVs. This pattern was corroborated by quantitative size binning analysis [Figure 2B], which showed increased relative abundance of EVs in the 100-150 nm range and decreased abundance in the > 200 nm fraction in the oxycodone group compared to controls. Consistent with the NTA profiles [Figure 2A], size-binning analysis [Figure 2B] demonstrated a shift toward smaller vesicles in the oxycodone group (increased 100-150 nm fraction with a corresponding decrease in the > 200 nm fraction); group comparisons were performed using an unpaired *t*-test with Welch’s correction, and significant differences are indicated on the figure (*P* < 0.05).

Complementing these results, particle concentration measurements [Figure 1F] demonstrated a significant increase in total EV count in the oxycodone group, suggesting an overall increase in EV biogenesis or release. These findings collectively indicate that chronic perinatal oxycodone exposure alters the physical properties of placental small EVs, potentially modifying their biological function and capacity for maternal-fetal signaling.

**Figure 3:**
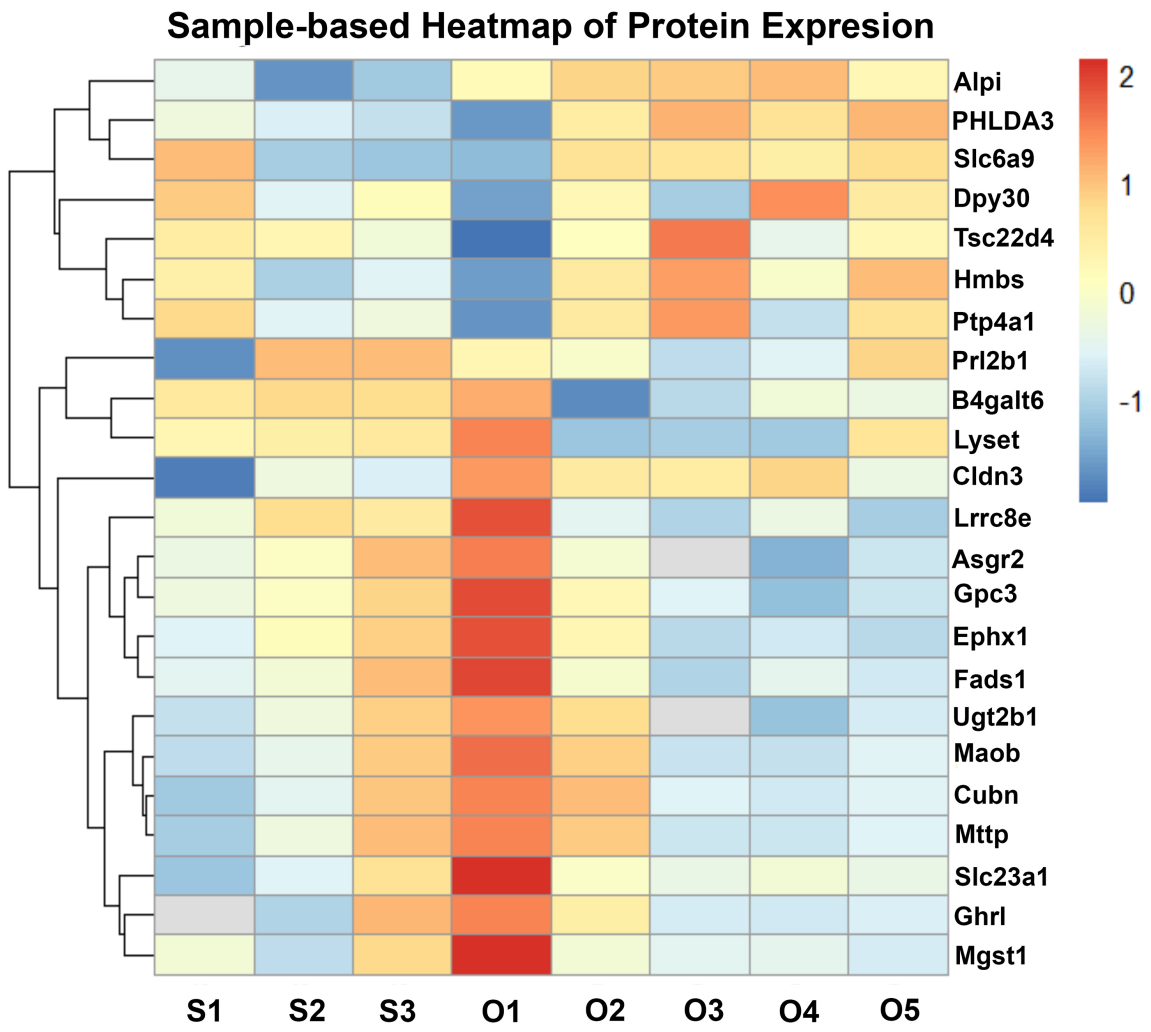
Oxycodone-induced changes in placental sEV protein expression. Sample-based heatmap showing z-score normalized protein expression across individual proteomics samples, with clear separation between saline and oxycodone groups. S indicates saline-derived EV samples (S1-S5) and O indicates oxycodone-derived EV samples (O1-O5); numeric suffixes denote individual biological replicate EV preparations included in the label-free proteomics analysis (not placenta numbering). Some within-group variability is observed, which is expected for biological replicates; however, overall clustering supports a consistent group-specific proteomic signature. sEV: Small extracellular vesicle.

To characterize the molecular alterations in placental EVs induced by chronic in utero oxycodone exposure, we performed label-free quantitative proteomic analysis followed by hierarchical clustering and differential expression profiling. In Figure 3, sample labels reflect individual biological replicate EV preparations (S = saline, O = oxycodone; e.g., S1-S5 and O1-O5), and the heatmap demonstrates clear separation between treatment groups. The sample-based heatmap [Figure 3] revealed consistent clustering of sEV protein expression patterns between oxycodone-exposed and saline-treated control samples, indicating reproducible group-specific proteomic signatures. A total of 456 proteins were identified in placental sEVs across all samples. Of these, 42 proteins were significantly upregulated, and 65 proteins were significantly downregulated in the oxycodone-exposed group compared to saline controls. Notably, several proteins, including histone methyltransferase complex regulatory subunit (Dpy30), homology-like domain family A member 3 (PHLDA3), solute carrier family 6 (neurotransmitter transporter, glycine), member 9 (Slc6a9), and TSC22 domain family member 4 (Tsc22d4), were consistently upregulated in oxycodone-exposed EVs. In contrast, proteins such as UDP glucuronosyltransferase 2 family, polypeptide B35 (Ugt2b35), carboxylesterase 1D (Ces1d1), microsomal glutathione S-transferase 1 (Mgst1), and monoamine oxidase B (Maob) were markedly downregulated across all oxycodone samples. Among key differentially expressed proteins (DEPs), the most consistently downregulated sEV cargo proteins in the oxycodone group were proteins involved in detoxification/oxidative stress defense and metabolic homeostasis. For example, microsomal glutathione S-transferase 1 (MGST1), which has been implicated in mitigating oxidative stress in trophoblasts and supporting trophoblast migration/invasion, was decreased in oxycodone-exposed sEVs. Likewise, UGT2B family members (UDP-glucuronosyltransferases), which contribute to xenobiotic/drug metabolism and detoxification, were reduced, consistent with impaired placental protective/metabolic capacity. In contrast, several upregulated EV proteins were consistent with cellular stress and altered signaling/trafficking, exemplified by PHLDA3, a p53-associated stress-response protein that is increased under hypoxic/stress conditions in placental tissues. At the pathway level, upregulated EV proteins were enriched for cytoplasmic translation initiation and vesicle trafficking/transport processes, whereas downregulated proteins were enriched for fatty acid degradation, TCA cycle/pyruvate metabolism, and N-linked glycosylation/N-glycan biosynthesis, supporting a shift toward stress-adaptive protein handling and away from core metabolic/biosynthetic functions.

### Functional enrichment of upregulated sEV proteins reveals altered translation and vesicle transport pathways

**Figure 4:**
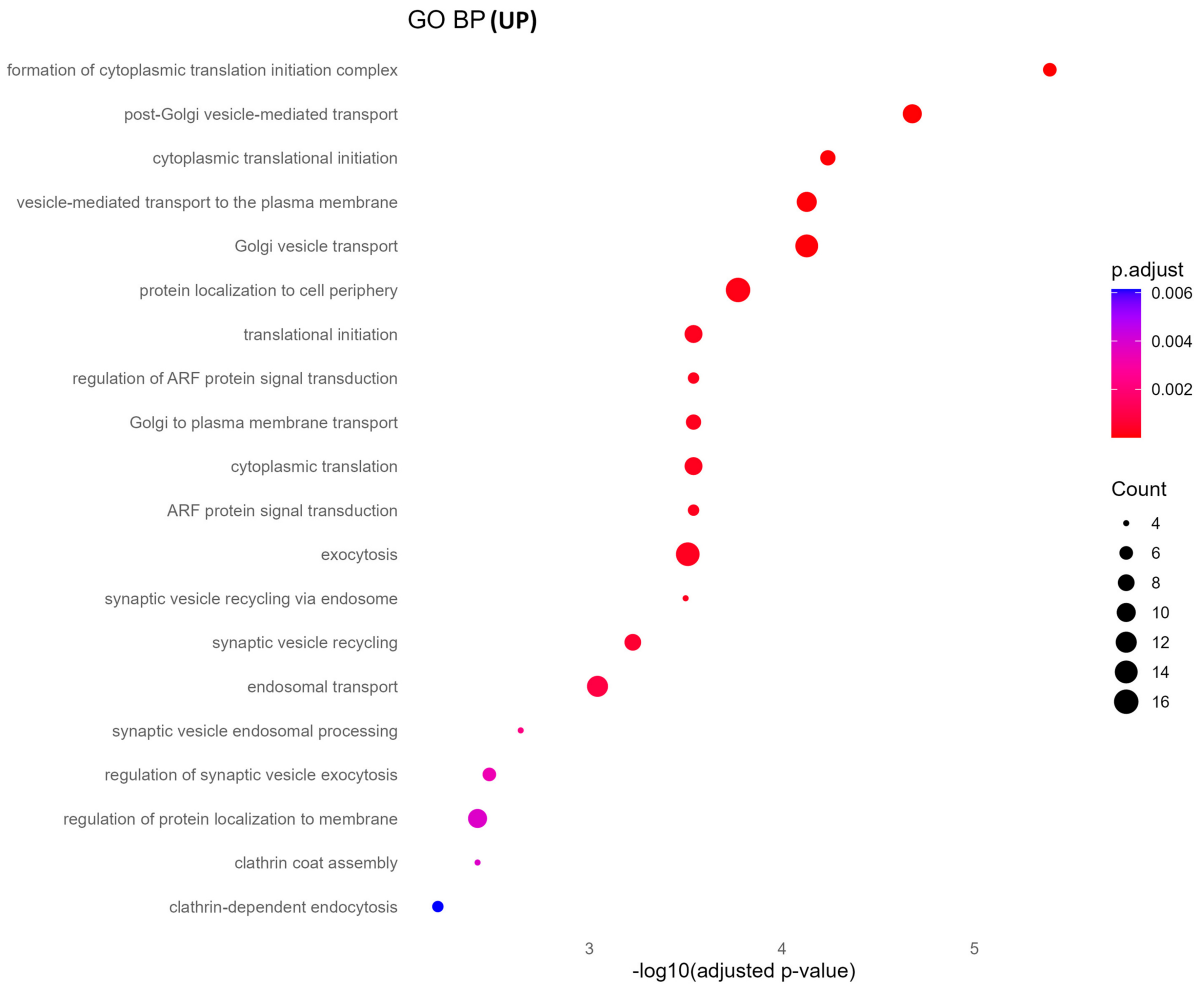
GO biological processes enrichment of upregulated proteins. This dot plot illustrates the top enriched GO biological processes in upregulated sEV proteins. Highly enriched terms include protein translation initiation, vesicle-mediated transport, and Golgi vesicle transport, suggesting enhanced protein synthesis and trafficking. Dot size and color encode gene ratio and -log10 *P*-value, respectively. GO: Gene Ontology; sEV: small extracellular vesicle; BP: biological process.

**Figure 5:**
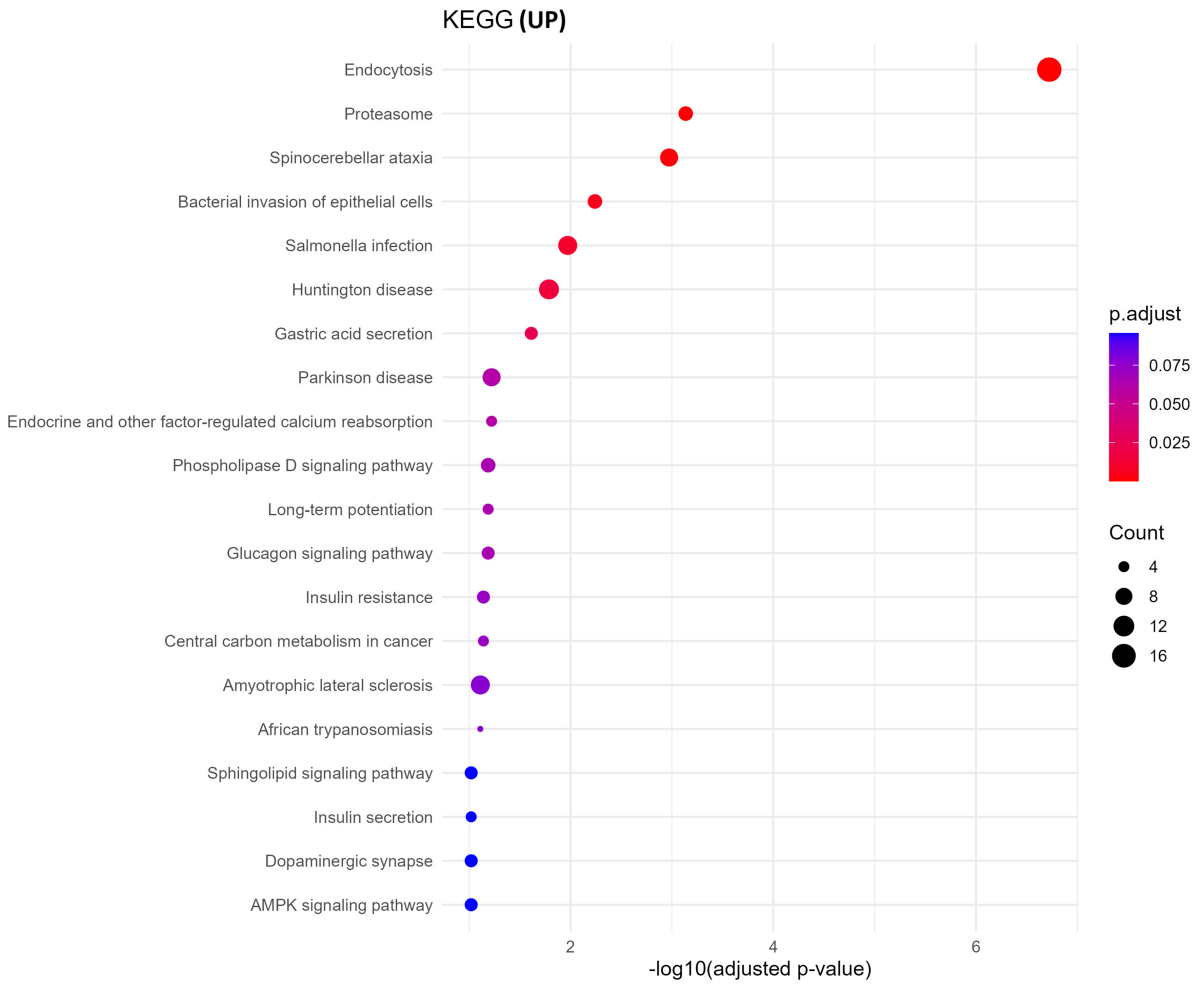
KEGG pathway enrichment of upregulated proteins. KEGG enrichment analysis of upregulated sEV proteins reveals pathways linked to endocytosis, proteasome activity, and neurodegenerative diseases such as Parkinson’s and Huntington’s. The size of each dot corresponds to the gene ratio, and the color gradient represents -log10 *P*-value. KEGG: Kyoto Encyclopedia of Genes and Genomes; sEV: small extracellular vesicle.

GO biological process analysis of upregulated proteins in oxycodone-exposed placental sEVs revealed significant enrichment in pathways related to protein synthesis and vesicle trafficking [Figure 4]. These include cytoplasmic translation initiation, synaptic vesicle recycling, Golgi-to-plasma membrane transport, and ADP-ribosylation factor (ARF) protein signal transduction. KEGG pathway enrichment corroborated these findings, identifying neurodegenerative disease pathways (e.g., Parkinson’s, Huntington’s, and spinocerebellar ataxia), proteasome function, and vesicle-mediated signaling processes as prominently altered [Figure 5]. These results suggest that oxycodone exposure induces compensatory or stress-related translational and trafficking adaptations within the placenta.

### Downregulated sEV proteins indicate disruption of metabolic and glycosylation pathways

**Figure 6:**
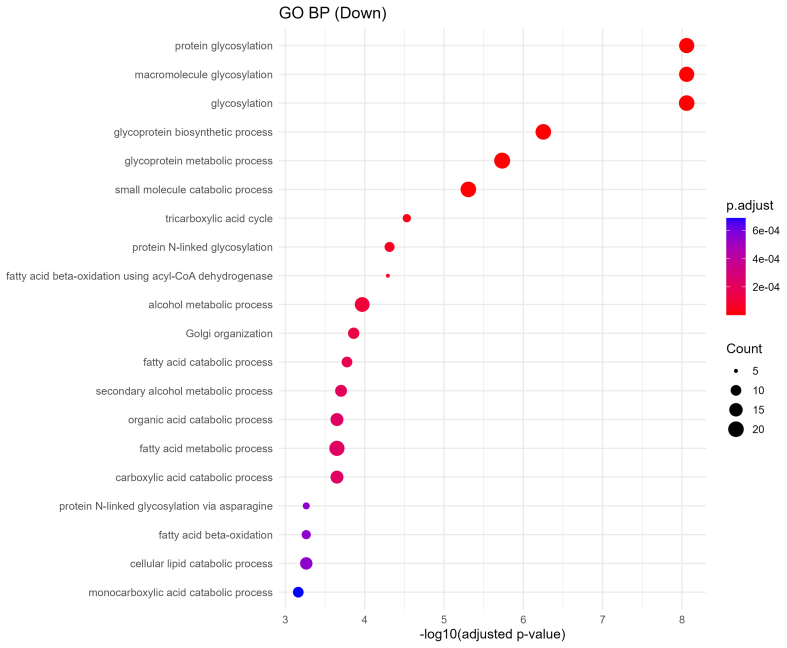
GO biological processes enrichment of downregulated proteins. Dot plot showing downregulated sEV protein-associated GO biological processes. Key disrupted processes include glycosylation, tricarboxylic acid cycle, fatty acid catabolism, and Golgi organization, indicating metabolic and glycoprotein-processing impairments. Dot size and color scale reflect gene ratio and statistical significance. GO: Gene Ontology; sEV: small extracellular vesicle; BP: biological process.

**Figure 7:**
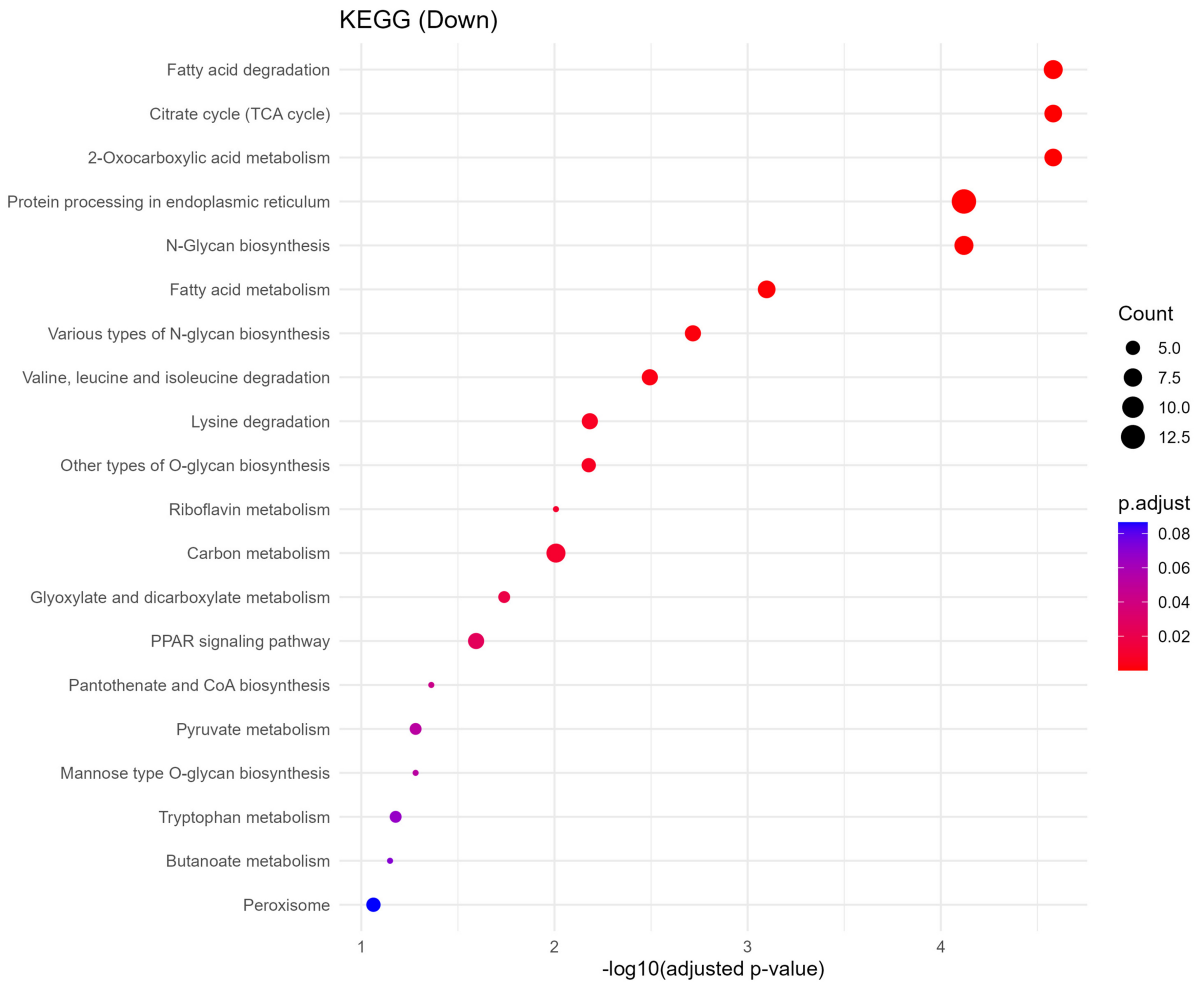
KEGG pathway enrichment of downregulated proteins. Dot plot displaying the top 20 significantly enriched KEGG pathways associated with downregulated sEV proteins. Notable pathways include fatty acid degradation, citrate cycle (TCA), and N-glycan biosynthesis, indicating disrupted energy metabolism and glycosylation. Dot size represents gene ratio, and color reflects -log10 *P*-value. KEGG: Kyoto Encyclopedia of Genes and Genomes; sEV: small extracellular vesicle; TCA: tricarboxylic acid.

Downregulated proteins were strongly enriched in metabolic processes critical to placental and fetal development. GO biological process terms included fatty acid degradation, citrate cycle (TCA cycle), pyruvate metabolism, and protein N-linked glycosylation via asparagine [Figure 6]. KEGG analysis further identified impairments in pathways such as amino acid metabolism, lysine degradation, N-glycan biosynthesis, and peroxisomal function [Figure 7]. These changes imply reduced energy production and impaired biosynthetic function, which may compromise fetal nutrient availability and cellular homeostasis.

### Oxycodone exposure suppresses cardiomyopathy-associated pathways in placental sEVs

**Figure 8:**
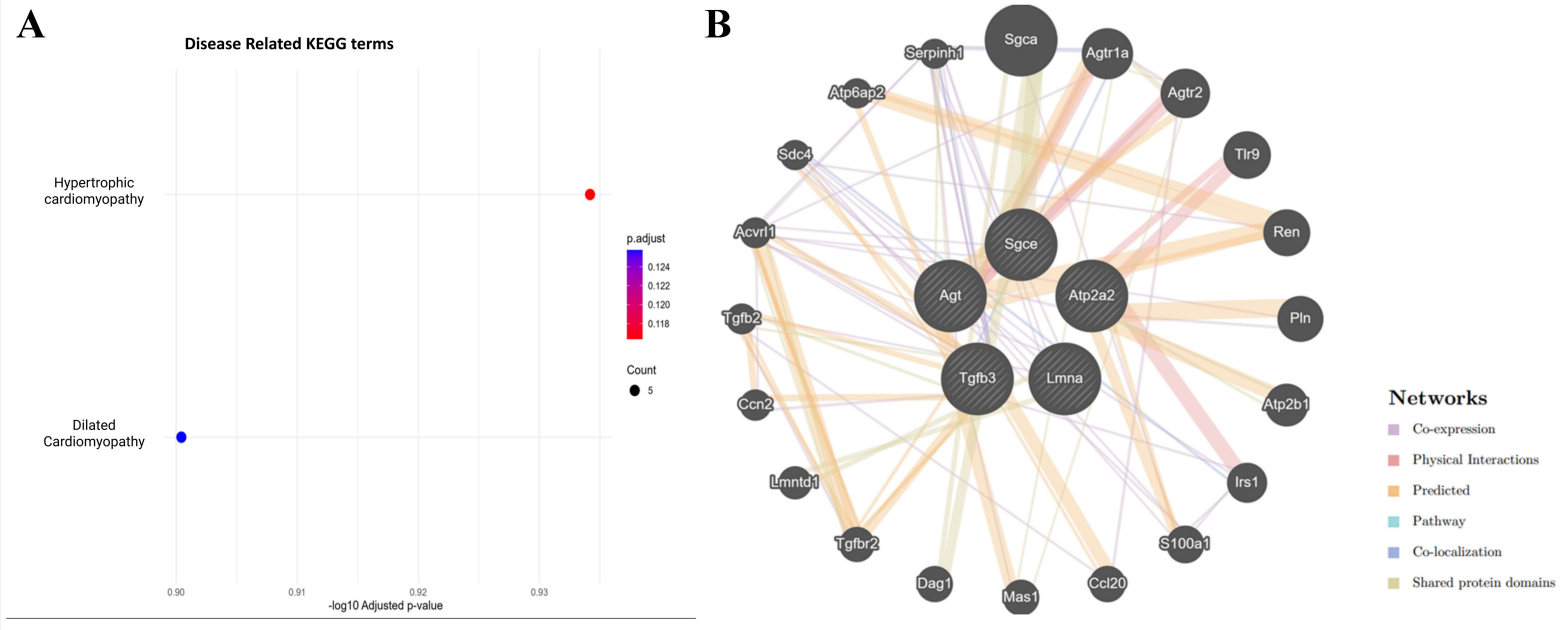
(A) Disease-specific KEGG enrichment shows significant downregulation of proteins involved in hypertrophic and DCM; (B) GeneMANIA interaction network of five downregulated cardiomyopathy-related proteins (Atp2a2, Lmna, Tgfb3, Agt, and Sgce) reveals interactions with regulators of cardiac signaling, fibrosis, calcium handling, and vascular function. KEGG: Kyoto Encyclopedia of Genes and Genomes; DCM: dilated cardiomyopathy.

Disease-targeted KEGG enrichment analysis revealed significant downregulation of proteins involved in hypertrophic cardiomyopathy (HCM) and DCM [Figure 8A]. Five proteins - Atp2a2, Lmna, Tgfb3, Agt, and Sgce - were consistently suppressed in oxycodone-treated sEVs. These proteins are known regulators of cardiac calcium handling, nuclear architecture, extracellular matrix remodeling, renin-angiotensin signaling, and membrane stabilization [Table 1]. Their downregulation highlights a potential mechanism by which perinatal opioid exposure may interfere with fetal cardiac development.

### GeneMANIA interaction network highlights cardiac and hormonal regulatory disruption in placental sEVs

To explore the broader regulatory landscape of the suppressed cardiomyopathy-related proteins, we constructed a GeneMANIA network incorporating Atp2a2, Lmna, Tgfb3, Agt, and Sgce [Figure 8B]. The resulting network revealed strong interactions with additional cardiac and endocrine regulators such as Renin (Ren), angiotensin II receptor, type 1a (Agtr1a), transforming growth factor, beta receptor II (Tgfbr2), Phospholamban (Pln), and Sarcoglycan, alpha (dystrophin-associated glycoprotein) (Sgca). These proteins are essential for calcium signaling, blood pressure regulation, myocardial remodeling, and hormonal control. The densely connected interaction map supports a model in which chronic oxycodone exposure disrupts a coordinated signaling hub essential for fetal cardiac and vascular development.

## DISCUSSION

In this study, we present the first comprehensive systems-level investigation of how chronic perinatal oxycodone exposure reshapes the molecular composition and biophysical properties of PSEVs. We found that prolonged opioid exposure disrupts EV-mediated signaling, altering their molecular cargo in ways that may predispose the fetus to systemic developmental vulnerabilities. Biophysical profiling using TEM and NTA revealed marked alterations in the size and morphology of PSEVs following oxycodone exposure [Figure 2A]. EVs from the oxycodone group exhibited significantly smaller average diameters compared to saline controls, with increased enrichment in the 100-150 nm range and a depletion of larger vesicles (> 200 nm) [Figure 2B]. These changes suggest stress-induced disruption in EV biogenesis or cargo packaging mechanisms^[22]^.

During gestation, large quantities of biomolecules are transported from the mother to the fetus via the placenta^[26]^. Often, this material is securely delivered to specific target areas through EVs. This essential function positions EVs as critical mediators in several in utero developmental processes. Trophoblast differentiation, brain and cardiomyocyte maturation, angiogenesis, and feto-maternal immune tolerance are key physiological events regulated by EVs^[27]^. Neurotoxic compounds, such as oxycodone, can significantly alter the molecular composition of EV cargo^[11]^. Both surface and internal DEPs within EVs are crucial for ensuring the adequate delivery of biological materials to the fetus throughout pregnancy. Using proteomics analysis, we observed significant shifts in the molecular profile of the oxycodone group’s DEPs.

MGST1 showed significant downregulation in sEV cargo from the oxycodone group. This protein has been reported to mitigate oxidative stress within the trophoblasts, playing a beneficial role in both trophoblast cell migration and invasion. These processes are necessary for proper placental development. Decreases in MGST1 have been associated with higher rates of pre-eclampsia complications during pregnancy^[28]^. UDP-glucuronosyltransferase (UGT), particularly UGT2B, has been recognized as a valuable protein coming from a “mammalian superfamily” of metabolizing enzymes, vital to removing toxic compounds from internal milieu^[29]^. The placenta plays a vital role in the detoxification, metabolism, and excretion of harmful substances, assisting in protecting the fetus from toxic materials. However, we observe decreased levels of UGT2B (particularly in sEVs) in the oxycodone-exposed group that may be associated with placental development^[30]^. These lower levels of vital detoxification proteins can allow for harmful drugs such as oxycodone to enter prominently into placental-fetal circulation^[29]^. Higher levels of PHLDA3 were found in sEV cargo from the oxycodone group. This DEP, which is activated by the p53 tumor suppressor pathway, is increased in placental tissues under hypoxic conditions^[31]^. The significant increase in PHLDA3 expression observed in the oxycodone group is consistent with a stress response.

Global enrichment analyses revealed that downregulated proteins were enriched in essential metabolic and structural pathways [Figure 6 and Supplementary Figure 1], including fatty acid degradation, the TCA cycle, pyruvate metabolism, and N-glycan biosynthesis. Given the crucial role of the placenta in nutrient exchange, these findings suggest impaired energy metabolism and biosynthetic functions in the fetus, potentially disrupting organ growth and development. Conversely, upregulated proteins were enriched in processes related to vesicle-mediated transport, cytoplasmic translation initiation, and neurodegenerative disease pathways, including Parkinson’s, Huntington’s, and spinocerebellar ataxia [Figure 5]. These alterations are indicative of compensatory stress responses or altered intercellular signaling in response to chronic oxycodone exposure [Supplementary Figure 2]. The affected pathways are representative of results seen in previous literature examining changes in sEVs following opioid exposure. Opioid use disorder (OUD) has been linked to alterations in neuroinflammatory mechanisms, and maternal inflammation has been shown to negatively impact fetal metabolic adaptation^[32,33]^.

Strikingly, one important facet we have found and that is not reported to date is oxycodone-associated placental effects possibly contributing to development of fetal cardiomyopathy. We employed a disease-targeted KEGG enrichment approach to determine whether specific pathological signatures were linked to the observed proteomic changes. Notably, we found significant enrichment of proteins involved in HCM and DCM [Figure 8A]. While our proteomic data strongly implicates downregulation of cardiomyopathy-associated proteins in placental sEVs following chronic oxycodone exposure, translating these findings into functional cardiac outcomes remains essential. For example, preclinical studies have found that prenatal stress or hypoxia (which similarly alter EV cargo) can lead to measurable reductions in fetal cardiac contractility, changes in cardiomyocyte size, and impaired calcium handling in offspring hearts^[34,35]^. Establishing whether the downregulation of proteins such as Atp2a2, Tgfb3, Lmna, Agt, and Sgce causes altered heart structure (e.g., ventricular wall thickness), or functional deficits (e.g., reduced ejection fraction, slower conduction, arrhythmias) in fetuses or neonates will strengthen the mechanistic link. Future experiments using echocardiography, histology, and assays of cardiac physiology (e.g., calcium imaging in isolated cardiomyocytes) in oxycodone exposed offspring will help determine how sEV proteome changes map to cardiac performance and risk of cardiomyopathy later in life. A study by Julian *et al.* found downregulation of Atp2a2 in placentas from preeclampsia cases, suggesting that dysfunction in ion channel expression plays a role in development of preeclampsia^[36]^. Furthermore, Chen *et al.* discovered a downregulation of Lmna in maternal serum collected from pregnancies with fetal CHD and preeclampsia^[37]^.

Our GeneMANIA interaction network [Figure 8B] showed strong relation between the previously mentioned cardiomyopathy-related proteins and other proteins such as Ren, Agtr1a, Tgfbr2, Pln, and Sgca. These proteins collectively orchestrate calcium signaling, blood pressure regulation, cardiac fibrosis, and extracellular matrix homeostasis^[38]^. Disruption of this network may impair fetal cardiac programming and increase postnatal susceptibility to cardiovascular disease^[39]^. Specifically, Tgfbr2 signaling has been shown to be inhibited in serum from preeclamptic pregnancies^[40]^.

Based on our data, one could hypothesize that chronic perinatal oxycodone exposure may lead to long-term adverse health outcomes in offspring. The downregulation of key cardiomyopathy-associated proteins suggests risk of structural heart issues (e.g., ventricular dilation or hypertrophy), impaired cardiac contractility, and susceptibility to cardiovascular disease later in life (e.g., heart failure or arrhythmias). Furthermore, metabolic pathway disruptions (e.g., in fatty acid oxidation, TCA cycle, glycosylation) might contribute to long-term metabolic dysfunction, such as insulin resistance, lipid dysregulation, or hepatic stress. These combined cardiac and metabolic insults could impair growth, exercise tolerance, and overall health trajectories in infants exposed to oxycodone in utero. Longitudinal studies following exposed offspring into infancy, childhood, and beyond will be essential to examine these hypotheses and assess the potential for early interventions.

Our findings in a rodent preclinical model provide valuable insights, yet several confounding factors limit direct extrapolation to human pregnancy. Notably, significant species differences exist in placental structure and function: rodents possess a labyrinthine placenta with distinct trophoblast organization and endocrine signaling compared to the human hemochorial placenta. Comparative reviews highlight differences in the timing and depth of trophoblast invasion, vascular remodeling, and hormonal regulation between species^[41]^. Additionally, oxycodone dosing, timing, and metabolic processing in rats differ from typical human exposure patterns, including variations in drug half-life and maternal–fetal pharmacokinetics. Other variables - such as maternal nutrition, stress, and co-exposures - may further influence both sEV release and fetal cardiac development. Recognizing these limitations and designing follow-up studies that better approximate human drug exposure, or that incorporate human placental tissues, will help determine which EV-associated proteomic alterations are conserved across species.

Collectively, these results demonstrate that chronic oxycodone exposure triggers multi-systemic molecular perturbations in placental sEVs, affecting cardiomyopathy-related proteins, essential metabolic networks, and vesicle trafficking functions. The altered sEVs profiles we identified may serve as powerful non-invasive biomarkers for assessing fetal risk in opioid-exposed pregnancies and provide a mechanistic link between maternal opioid use and altered fetal organogenesis^[14]^. Identifying alterations in placental sEV cargo as potential biomarkers is promising, yet several important limitations must be addressed before clinical translation. In humans, maternal blood–derived sEVs originate from multiple tissues - including placenta, maternal organs, and immune cells - so the specificity of cardiomyopathy-associated protein changes to placental sEVs requires rigorous validation. Timing also represents a key consideration: in our study, sEVs were collected at a single developmental stage (postnatal day 21 in rats), whereas human gestation encompasses multiple dynamic windows during which sEV composition likely shifts. Additionally, genetic diversity, environmental exposures, and comorbid conditions such as preeclampsia, diabetes, and maternal obesity may influence sEV cargo profiles in human populations. Thus, before these markers can be used for prenatal risk stratification, large-scale clinical cohort studies with serial maternal blood sampling - paired with detailed fetal and neonatal cardiac outcomes - are needed to establish sensitivity, specificity, and predictive utility.

Despite the novelty and strengths of our approach, several limitations warrant consideration. First, the modest sample size may limit statistical power and reduce generalizability. Second, the cross-sectional nature of our sEV sampling prevents evaluation of temporal changes in EV cargo across gestation. Third, although the rat model provides valuable mechanistic insight, interspecies differences in placental architecture, drug metabolism, and EV biogenesis complicate direct extrapolation to human pregnancy. Finally, potential off-target effects of oxycodone or unmeasured environmental factors cannot be fully excluded. These limitations highlight the need for cautious interpretation and underscore the importance of validating our findings in larger, longitudinal, and clinically relevant cohorts.

Future studies would extend these findings to human placental sEVs, evaluate temporal changes in sEVs cargo during gestation, and assess long-term cardiovascular outcomes in offspring. Several recent studies have begun to characterize sEVs in human pregnancy, with emerging clinical data supporting their utility as biomarkers for placental dysfunction, preeclampsia, and fetal growth restriction^[42]^. However, the impact of opioid exposure on sEVs profiles in human cohorts remains underexplored. Incorporating high-throughput sEVs profiling into longitudinal birth cohorts and obstetric clinical studies could further help validate these molecular signatures and the overall translational relevance of PSEV-targeted interventions to mitigate opioid-induced fetal cardiac risk. In conclusion, our findings demonstrate that chronic perinatal oxycodone exposure significantly alters the composition and function of PSEVs, revealing a previously underappreciated mechanism by which opioid use during pregnancy may disrupt fetal development. The downregulation of cardiomyopathy-associated proteins and metabolic regulators within sEVs suggests that oxycodone exposure impairs key signaling pathways essential for cardiac structure, calcium homeostasis, and energy metabolism. These changes highlight the placenta-sEV axis as a critical conduit for opioid-induced developmental risk and position sEVs as promising non-invasive biomarkers for assessing fetal cardiovascular vulnerability. Collectively, this study underscores the need to further explore the diagnostic and therapeutic potential of placental sEVs in the context of maternal opioid use and fetal health.

## DECLARATIONS

### Availability of data and materials

The MS proteomics raw data have been deposited to the ProteomeXchange Consortium via the PRIDE partner repository with the dataset identifier PXD065451. All other relevant data supporting the key findings of this study are available within the article and its Supplementary Materials.

### Copyright

© The Author(s) 2026.
