# Supplementary material for: Chronic in utero oxycodone exposure alters placental small EV proteome and fetal cardiomyopathy-linked pathways
Source: Extracell Vesicles Circ Nucl Acids. 2026 Feb 10;7(1):146–64. doi: 10.20517/evcna.2025.138 (PMC13074287; doi:10.20517/evcna.2025.138)
Supplement: Supplementary file 1 [file evcna-7-1-146-SupplementaryMaterials.zip › evcna60138-Supplementary Figures.pdf]

Supplementary Materials

Chronic in utero oxycodone exposure alters placental small EV proteome and fetal cardiomyopathy-linked pathways

Amin Foroughi-Nezhad[1,#], Dalia Moore[1,#], Victoria L. Schaal[1], Tousif Ahmed Hediyal[1], Elizabeth Stone[1], Sree Kolli[1], Pranavi Athota[1], Omar Shukri[1], Sowmya V. Yelamanchili[1,2], Gurudutt Pendyala[1,2,3,4]

[1]Department of Anesthesiology, College of Medicine, University of Nebraska Medical Center (UNMC), Omaha, NE 68198, USA.

[2]Department of Genetics, Cell Biology, and Anatomy, College of Medicine, University of Nebraska Medical Center (UNMC), Omaha, NE 68198, USA.

[3]Child Health Research Institute, University of Nebraska Medical Center (UNMC), Omaha, NE 68198, USA.

[4]National Strategic Research Institute, University of Nebraska Medical Center (UNMC), Omaha, NE 68198, USA.

[#]These authors contributed equally to this work.

Correspondence to: Dr. Gurudutt Pendyala, Dr. Sowmya V. Yelamanchili, Department of Anesthesiology, College of Medicine, University of Nebraska Medical Center (UNMC), Omaha, NE 68198, USA. E-mail: gpendyala@unmc.edu; syelamanchili@unmc.edu

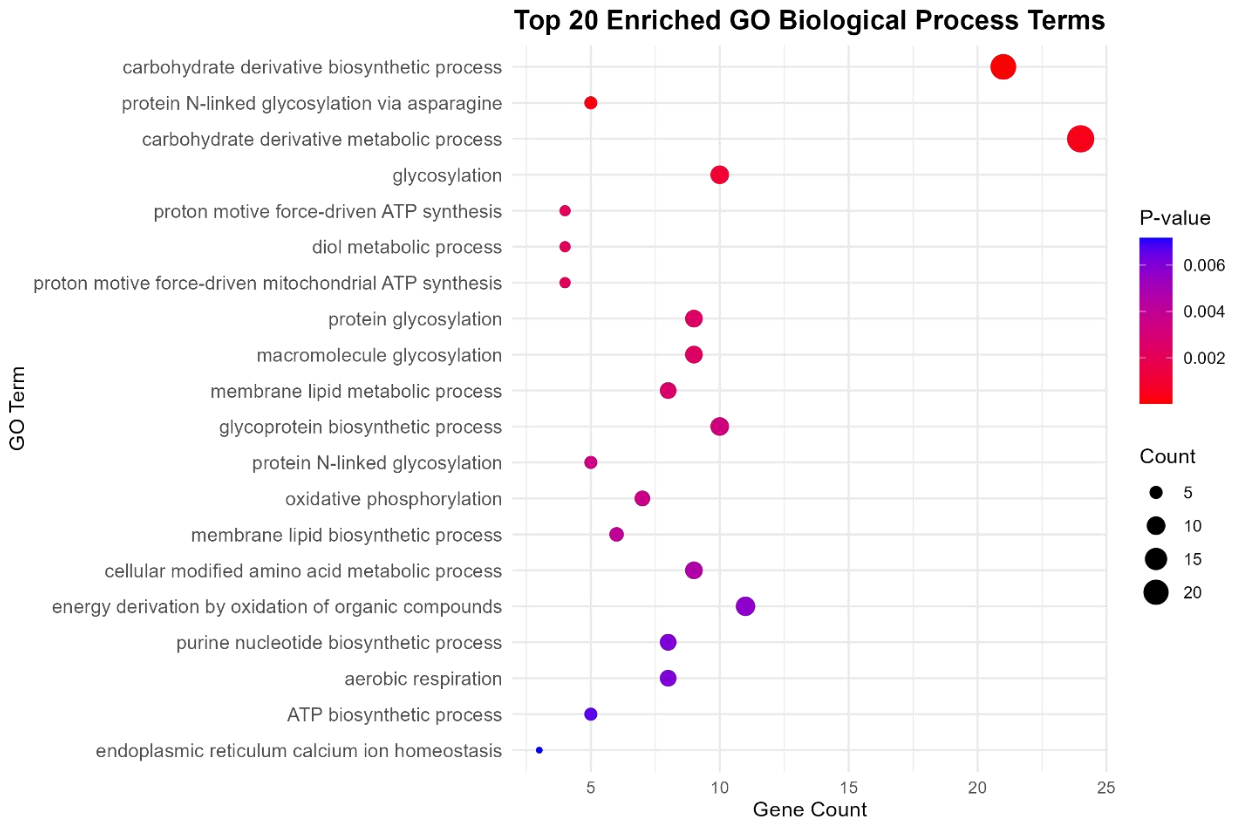

Supplementary Figure 1. (GO Biological Processes):

This bubble plot shows the Holistic Gene Ontology (GO) enrichment analysis of significant proteins.

- Y-axis: GO Biological Process terms.
- X-axis: Gene counts associated with each term.
- Bubble Size: Number of proteins involved in the process.
- Bubble Color: Significance level (*p*-value), with red indicating highly significant terms and blue less significant ones.
- Top Results: Highlight processes like *carbohydrate derivative biosynthetic process*, *glycosylation*, and *oxidative phosphorylation*.

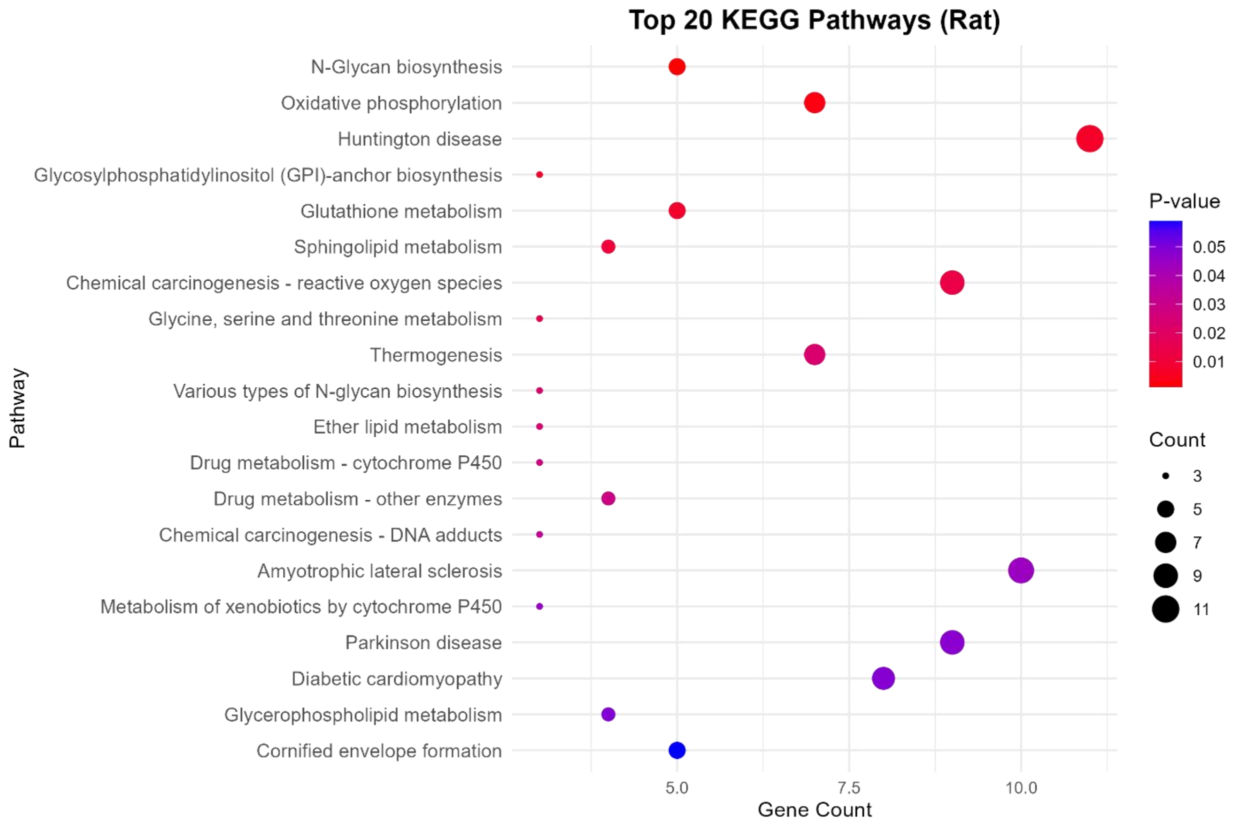

Supplementary Figure 2. (KEGG Pathways):

This bubble plot depicts Holistic KEGG pathway enrichment analysis of the same significant proteins.

- Y-axis: Enriched KEGG pathways.
- X-axis: Gene counts associated with each pathway.
- Bubble Size: Number of proteins contributing to each pathway.
- Bubble Color: *p*-value significance, with red being most significant.
- Top Pathways: Include *N-Glycan biosynthesis*, *Oxidative phosphorylation*, and *Huntington disease*.
